# Identification of vital candidate microRNA/mRNA pairs regulating ovule development using high-throughput sequencing in hazel

**DOI:** 10.1186/s12861-020-00219-z

**Published:** 2020-07-01

**Authors:** Jianfeng Liu, Qizheng Luo, Xingzheng Zhang, Qiang Zhang, Yunqing Cheng

**Affiliations:** Jilin Provincial Key Laboratory of Plant Resource Science and Green Production, Jilin Normal University, Siping, Jilin Province 136000 PR China

**Keywords:** microRNA, mRNA, Transcriptome, Ovule, Development, Hazel

## Abstract

**Background:**

Hazels (*Corylus* spp.) are economically important nut-producing species in which ovule development determines seed plumpness, one of the key parameters reflecting nut quality. microRNAs (miRNAs) play important roles in RNA silencing and the post-transcriptional regulation of gene expression. However, very little is currently known regarding the miRNAs involved in regulating ovule growth and development.

**Results:**

In this study, we accordingly sought to determine the important miRNAs involved in ovule development and growth in hazel. We examined ovules at four developmental stages, namely, ovule formation (Ov1), early ovule growth (Ov2), rapid ovule growth (Ov3), and ovule maturity (Ov4). On the basis of small RNA and mRNA sequencing using the Illumina sequencing platform, we identified 970 miRNAs in hazel, of which 766 and 204 were known and novel miRNAs, respectively. In Ov1-vs-Ov2, Ov1-vs-Ov3, Ov1-vs-Ov4, Ov2-vs-Ov3, Ov2-vs-Ov4, and Ov3-vs-Ov4 paired comparisons, 471 differentially expressed microRNAs (DEmiRNAs) and their 3117 target differentially expressed messenger RNAs (DEmRNAs) formed 11,199 DEmiRNA/DEmRNA pairs, with each DEmiRNA changing the expression of an average of 6.62 target mRNAs. Kyoto Encyclopedia of Genes and Genomes (KEGG) enrichment analysis of all DEmRNAs revealed 29 significantly enriched KEGG pathways in the six paired comparisons, including protein export (ko03060), fatty acid elongation (ko00062), starch and sucrose metabolism (ko00500), fatty acid biosynthesis (ko00061), and amino sugar and nucleotide sugar metabolism (ko00520). Our results indicate that DEmiRNA/DEmRNA pairs showing opposite change trends were related to stress tolerance, embryo and seed development, cell proliferation, auxin transduction, and the biosynthesis of proteins, starch, and fats may participate in ovule growth and development.

**Conclusions:**

These findings contribute to a better understanding of ovule development at the level of post-transcriptional regulation, and lay the foundation for further functional analyses of hazelnut ovule growth and development.

## Background

Hazels (*Corylus* spp.) are important nut-producing species in the Betulaceae family (order: Fagales), the nut kernel of which is an important raw material in food processing industries [1]. After shelling, plump hazel kernels are generally processed as toasted kernels due to their desirable appearance, whereas less well developed and shriveled kernels are processed for oil, powder, jam, and kernel crumb. Seed plumpness is, therefore, an important parameter reflecting nut quality and is a major contributor to the market prices of these nuts. A better understanding of ovule development and filling is essential for high-quality kernel formation in hazel breeding and growth.

During nut formation, the ovule undergoes a complex series of developmental events. At anthesis of the pistillate inflorescences, the gynoecium is far from mature owing to the absence of a complete ovary and ovule [2, 3]. Only after pollination and extension of the pollen tube to the stigma, do the ovary and ovule primordium begin to differentiate and form gradually within approximately 50 days. Consequently, fertilization of the ovule and embryo sac maturation tend to take considerably longer in hazelnut than in most angiosperms [2, 3]. Our previous comparative transcriptome analysis showed that genes related to auxin biosynthesis, transport, signaling, the floral quartet model, and flower development may regulate ovary formation and fertilization in hazel [4, 5]. Interestingly, there are initially two ovules in the ovary of hazel, and although both of these ovules are fertilized, generally only one will develop into an edible kernel [6]. Although ovule size gradually increases from fertilization to maturity, the detailed molecular mechanisms underlying the regulation of ovule growth during the course of development have yet to be fully elucidated.

microRNAs (miRNAs) are small non-coding RNA molecules ranging from 18 to 24 nucleotides in length that occur extensively in plants, animals, and certain viruses, and play important roles in mRNA cleavage, RNA silencing and the post-transcriptional regulation of gene expression [7–10]. In plant cells, miRNAs typically undergo complementary interaction with target mRNA sequences, thereby disrupting transcription and silencing the target mRNA [9]. For example, in rice, the overexpression of the miRNA OsmiR397 results in an increase in grain size and promotes panicle branching via downregulation of its target gene *OsLAC*, which encodes a laccase-like protein involved in the sensitivity of plants to brassinosteroids. As a consequence, there is an increase in overall grain yield of 25% [11]. Similarly, miR156 has been demonstrated to regulate grain size, shape, and quality via silencing of its target genes *SPL14* and *SPL16* in rice [12–14]. Furthermore, in tomato (*Solanum lycopersicum*), miR168 regulates phase transition, leaf epinasty, and fruit development via regulation of its target gene *SlAGO1* [15], whereas overexpression of miR167 has been found to induce the silencing of *ARF8*, thereby producing the parthenocarpy trait in both *Arabidopsis* and tomato [16]. Due to specificity and fewer off-target effects, microRNA-based strategies are regarded as more efficient than those based on small interfering RNA [17]. Hence, the identification miRNAs and their target genes is considered as a preferable approach for gaining a better understanding of plant development and presents new opportunities for plant trait improvement.

Genome-wide analysis of miRNAs and their target genes can provide valuable insights into miRNA-based regulatory mechanisms in plants [18]. To date, however, comparatively little information has been obtained regarding miRNAs and their gene targets in hazel. Accordingly, in the present study, we used high-throughput sequencing technology to investigate the regulatory roles of miRNAs in hazel ovule development and to identify miRNAs, and mRNAs contributing to ovule development at four discrete developmental stages. Our comparison of miRNA and mRNA expression patterns at these developmental stages provides novel important insights into the molecular mechanisms underlying ovary development and growth in hazel.

## Results

### Overview of small RNA sequencing

**Fig. 1:**
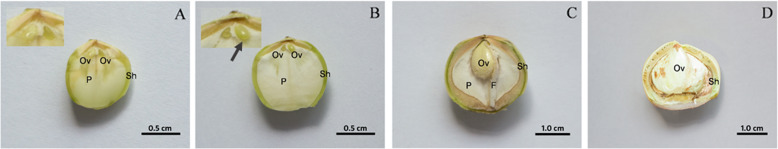
Fruit and ovule characteristics at four developmental stages. **a** Stage Ov1 (stage of ovule formation), two ovules in ovary are about equal in size; **b** Stage Ov2 (stage of early ovule growth), the diameter of large ovules is about two times the diameter of small ovules, arrow shows the large ovule. **c** Stage Ov3 (stage of rapid ovule growth), developing ovule accounted for about 60% of its full size; **d** Stage Ov4 (stage of ovule maturity), ovule develops to its full size. Key: P, parenchyma; Sh, shell; Ov, ovule; F, funiculus

In total, we obtained 201.1 million raw reads through Illumina sequencing of 12 small RNA libraries for ovules at four successive developmental stages (Ov1, Ov2, Ov3, and Ov4) (Fig. 1A, B, C and D), and they were analyzed to identify miRNAs. For each sample, the number of retrieved raw reads ranged between 12.3 and 26.0 million (Table 1), which provided sufficient sequencing data for the discovery of an extensive range of small regulatory RNAs. In each sample, we focused on reads with length between 18 and 30 nt, and these reads were mapped to public databases [Silva, GtRNAdb (a genomic tRNA database), Rfam, and Repbase] (Table 2) to annotate the composition of the small RNA populations. Among the annotated RNAs, we found rRNAs to be more abundant then snRNAs, snoRNAs, or tRNAs, accounting for 27.99% of the total reads, whereas unannotated RNAs accounted for 70.56% of the total reads, a proportion that is considerably higher than that of the annotated RNAs (Table 2).

**Table 1:** Summary statistics of small RNA sequencing

Samples	Raw reads	Containing ‘N’ reads	Length < 18	Length > 30	Clean reads	Q30 (%)
Ov1A	13,894,831	42	698,763	512,508	12,683,518	98.91
Ov1B	13,878,499	30	976,962	374,323	12,527,184	98.73
Ov1C	13,421,035	24	1,830,615	448,779	11,141,617	97.9
Ov2A	20,896,494	1001	2,142,443	1,523,358	17,229,692	99.47
Ov2B	19,445,202	803	5,886,080	660,526	12,897,793	99.41
Ov2C	26,021,769	1182	3,453,394	922,411	21,644,782	99.58
Ov3A	15,103,004	10	2,099,924	497,300	12,505,770	98.33
Ov3B	12,263,158	16	316,899	910,484	11,035,759	98.62
Ov3C	12,852,836	20	292,319	608,914	11,951,583	98.73
Ov4A	15,411,252	12	381,934	1,661,549	13,367,757	98.52
Ov4B	17,421,423	9	2,071,014	721,340	14,629,060	98.66
Ov4C	20,527,758	8	7,073,528	291,669	13,162,553	98.61
Total	201,137,261	3157	27,223,875	9,133,161	164,777,068	98.79

Note: Raw reads: Sequencing raw reads; Containing ‘N’ reads: reads containing at least 10% unknown base; Length < 18: reads less than 18 nucleotides after removing the adapter; Length > 30: reads greater than 30 nucleotides after removing the adapter; Clean_reads: Reads with a mass value greater than or equal to 30 bases; Q30 (%): bases proportion with a quality value greater than Q30

**Table 2:** Statistics summary of small RNA annotation

Samples	Clean_reads	rRNA	snRNA	snoRNA	tRNA	Repbase	Unannotated
Ov1A	12,683,518 (100.00%)	2,656,487 (20.94%)	0 (0.00%)	4189 (0.03%)	49,625 (0.39%)	6701 (0.05%)	9,966,516 (78.58%)
Ov1B	12,527,184 (100.00%)	2,775,092 (22.15%)	1 (0.00%)	4228 (0.03%)	44,277 (0.35%)	6702 (0.05%)	9,696,884 (77.41%)
Ov1C	11,141,617 (100.00%)	3,637,501 (32.65%)	0 (0.00%)	4091 (0.04%)	40,670 (0.37%)	6319 (0.06%)	7,453,036 (66.89%)
Ov2A	17,229,692 (100.00%)	3,935,661 (22.84%)	0 (0.00%)	42,156 (0.24%)	430,011 (2.50%)	15,635 (0.09%)	12,806,229 (74.33%)
Ov2B	12,897,793 (100.00%)	2,731,975 (21.18%)	2 (0.00%)	39,227 (0.30%)	305,727 (2.37%)	19,391 (0.15%)	9,801,471 (75.99%)
Ov2C	21,644,782 (100.00%)	4,207,691 (19.44%)	0 (0.00%)	50,788 (0.23%)	404,254 (1.87%)	18,606 (0.09%)	16,963,443 (78.37%)
Ov3A	12,505,770 (100.00%)	3,726,345 (29.80%)	0 (0.00%)	2458 (0.02%)	131,987 (1.06%)	5250 (0.04%)	8,639,730 (69.09%)
Ov3B	11,035,759 (100.00%)	2,947,478 (26.71%)	1 (0.00%)	1340 (0.01%)	179,619 (1.63%)	2090 (0.02%)	7,905,231 (71.63%)
Ov3C	11,951,583 (100.00%)	2,187,876 (18.31%)	0 (0.00%)	1583 (0.01%)	174,552 (1.46%)	3021 (0.03%)	9,584,551 (80.19%)
Ov4A	13,367,757 (100.00%)	4,844,278 (36.24%)	0 (0.00%)	1191 (0.01%)	144,594 (1.08%)	2226 (0.02%)	8,375,468 (62.65%)
Ov4B	14,629,060 (100.00%)	6,913,343 (47.26%)	2 (0.00%)	2142 (0.01%)	107,481 (0.73%)	3720 (0.03%)	7,602,372 (51.97%)
Ov4C	13,162,553 (100.00%)	5,559,768 (42.24%)	0 (0.00%)	2637 (0.02%)	114,188 (0.87%)	10,763 (0.08%)	7,475,197 (56.79%)
Total	164,777,068 (100%)	46,123,495 (27.99%)	6 (0.00%)	156,030 (0.09%)	2,126,985 (1.29%)	100,424 (0.06%)	116,270,128 (70.56%)

Note: *snRNA* small nuclear RNA; *snoRNA* small nucleolar RNA; *Repbase* repetitive elements database; unannotated, reads without match

### Prediction, length distribution, and first-base frequency of miRNAs

**Fig. 2:**
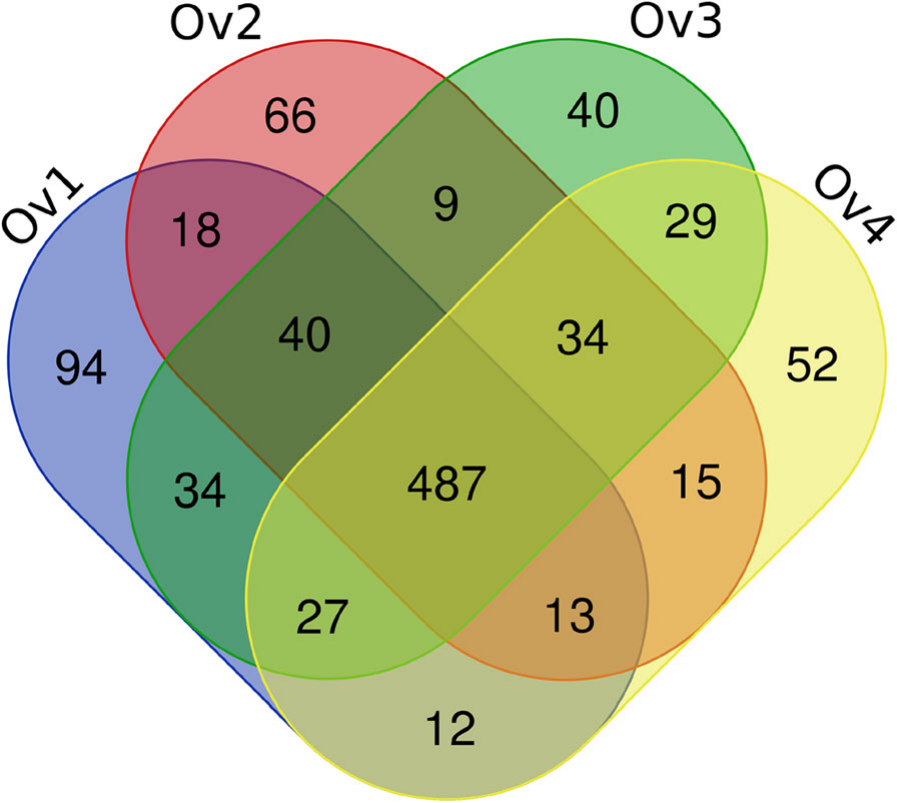
Venn diagram of all identified miRNA at four developmental stages

In order to discover novel miRNAs, we used the miRDeep2 software package with modifications for plant miRNAs to analyze the unannotated reads [19]. We accordingly identified 521, 682, 700, and 669 known miRNAs and 204, 204, 203, and 204 novel miRNAs in ovule samples collected at stages Ov1, Ov2, Ov3, and Ov4, respectively (Fig. 2; Table S1). In total, we identified 970 miRNAs in hazel, of which 766 were known miRNAs and 204 were novel miRNAs (Table S1). Whereas we detected 284 known miRNAs that were common to all four stages, 94, 66, 40, and 52 of the known miRNAs identified in Ov1, Ov2, Ov3, and Ov4 stages, respectively, were found to be stage unique (Table S1). On the basis of sequence similarity, the known and novel miRNAs were analyzed for miRNA family classification, and we accordingly found that the 970 miRNAs could be assigned to 165 families (Table S2). Among these families, we found that the highest number of identified miRNAs (60) were assigned to the MIR159 family, followed by the MIR166 (56), MIR171_1 (40), MIR482 (38), MIR396 (37), and MIR167_1 (34) families. Among all identified miRNAs, 951 had 18,137 responding target mRNAs, with individual miRNAs having an average of 18.70 target mRNAs (Table S3).

**Fig. 3:**
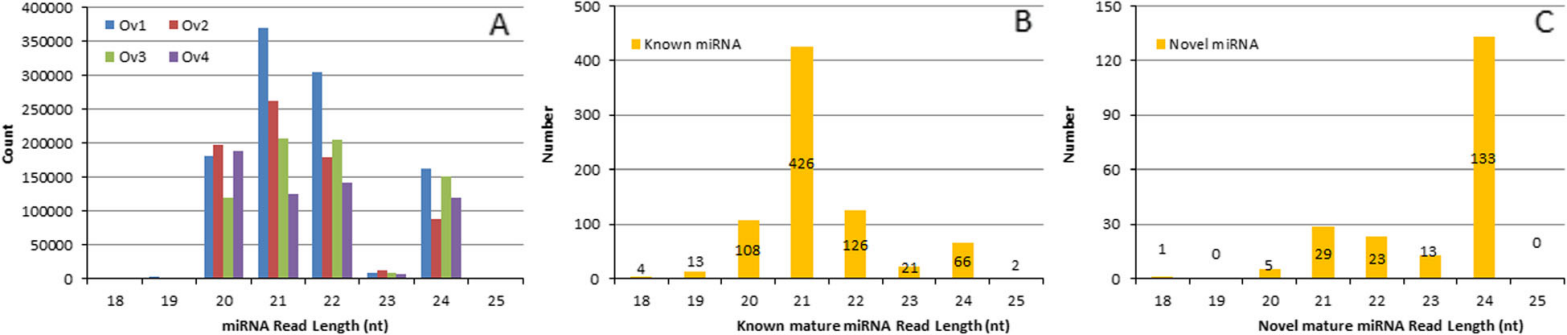
Characteristics of identified mature miRNAs in hazel. **a** miRNA reads count with different length; **b** Length distribution of known mature miRNAs; **c** Length distribution of novel mature miRNAs

Due to the specificity of the Dicer enzyme and the DICER-LIKE (DCL) enzyme, the final length of mature miRNA is mainly in the range of 20 nt to 24 nt, of which 21 nt or 24 nt are the main miRNAs in plants [18]. In total, we detected 3,045,973 reads in the 12 small RNA libraries, ranging in size from 18 to 25 nt. Among these, we obtained 1,029,270, 743,400, 690,516, and 582,787 small RNA read counts at stages Ov1, Ov2, Ov3, and Ov4, respectively (Table S4), showing a reduction in read count with ovule development. Most of the small RNA reads are 20 nt, 21 nt, 22 nt, and 24 nt in length (Fig. 3A). In terms of the length distribution of mature miRNAs, we found that miRNAs of 21 and 24 nt were the most abundant, followed by those of 22 and 20 nt, which collectively accounted for 94.43% of all the identified miRNAs (Fig. 3B and C). Notably, 133 of the 24-nt miRNAs were found to be novel, and accounted for 65.51% of all novel miRNAs (Fig. 3C). The correlation between miRNA samples reflects the similarity between gene expression in the samples. As the correlation coefficient reaches unity, the samples become more similar, and hence, the difference in miRNA gene expression is reduced. Correlation coefficients of genes in ovules of the same developmental stage in the current study are all close to unity, and from two adjacent stages are higher than samples from two non-adjacent stages (Fig. S1). These results also indicate that repeatability and homogeneity of the samples is good.

**Fig. 4:**
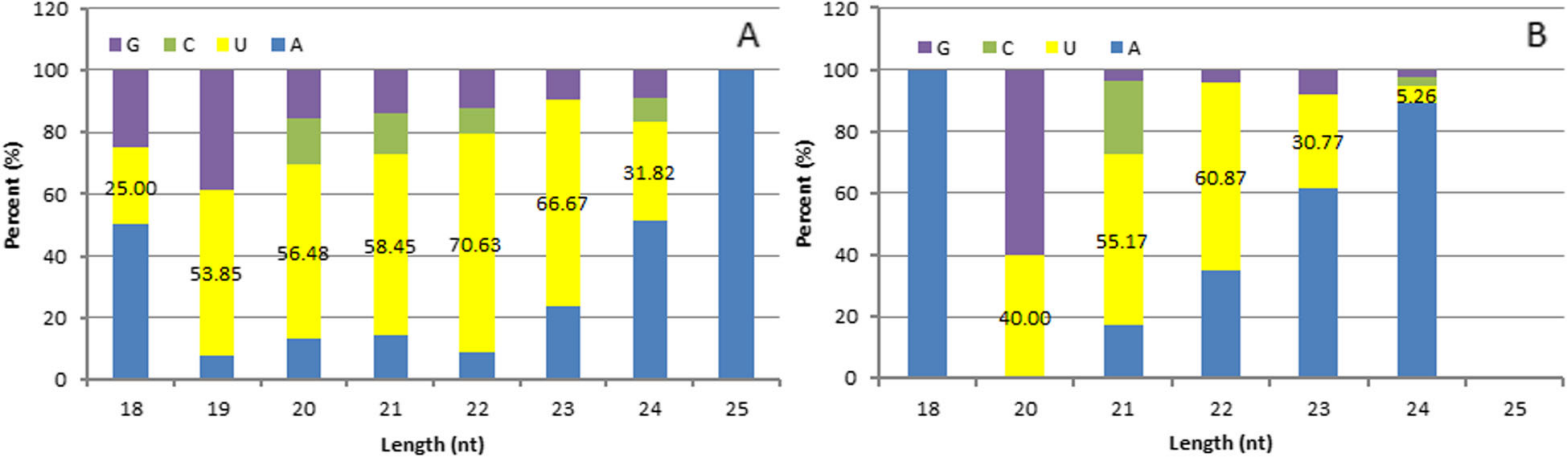
First base distribution of 18 nt to 25 nt miRNAs **a** First base distribution of 18 nt to 25 nt known miRNAs; **b** First base distribution of 18 nt to 25 nt novel miRNAs

Dicer enzyme or DCL is necessary for the formation of mature microRNA (miRNA), and it cleaves pre-miRNA correctly to generate mature miRNA with the correct seed regions [20]. When Dicer enzyme or DCL recognizes and cleaves the precursor miRNA, the first base of its 5′ terminal has a strong bias to uridine (U) [21]. Through the analysis of base preferences of miRNA, the typical base proportion of miRNA was obtained. On the basis of our determination of the first-base frequency of mature known and novel miRNAs (Fig. 4A and B), we found that among the known miRNAs, the 19–24-nt miRNAs preferentially started with a U, with percentages ranging from 31.82 to 70.63%. Similarly, for the novel miRNAs, we found that 20–23-nt miRNAs preferentially started with “U,” with percentages ranging from 30.77 to 60.87%.

### Identification of DEmiRNAs and their differentially expressed target mRNAs

**Fig. 5:**
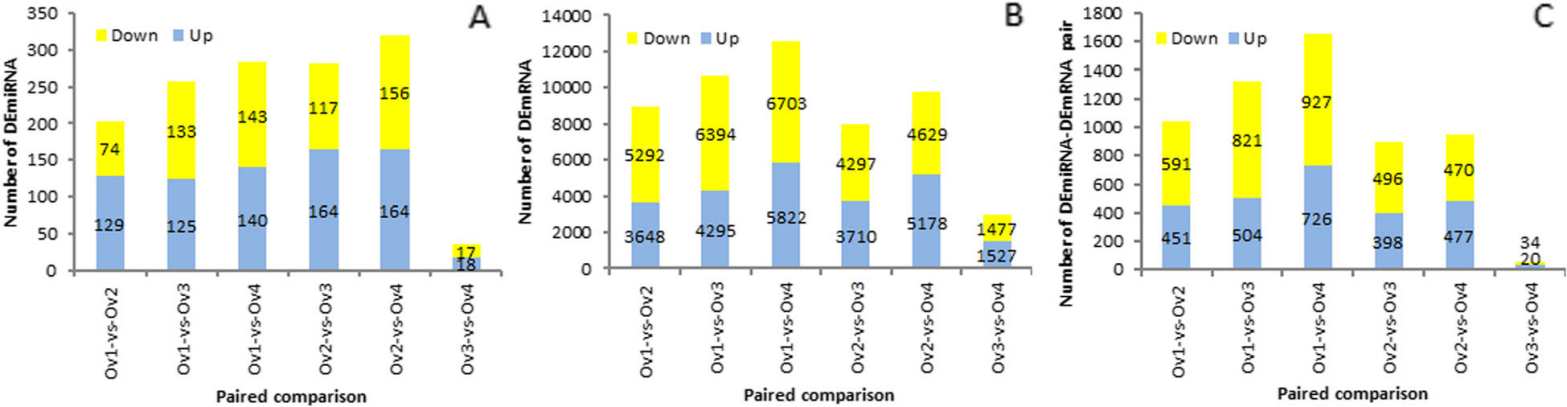
Statistical results of differentially expressed miRNAs (DEmiRNAs) and differentially expressed mRNAs (DEmRNAs). **a** Number of DEmiRNAs of six paired comparison; **b** Number of DEmRNAs of six paired comparison; **c** Number of target DEmRNAs of DEmiRNAs

DEmiRNAs among the six paired sample groups were identified. For the paired comparisons Ov1-vs-Ov2, Ov1-vs-Ov3, Ov1-vs-Ov4, Ov2-vs-Ov3, Ov2-vs-Ov4, and Ov3-vs-Ov4, we identified 203, 258, 283, 281, 320, and 35 DEmiRNAs, respectively (Fig. 5A), indicating the longer the interval between the two sample groups, the higher was the number of generated DEmiRNAs. In total, we detected 471 DEmiRNAs among the six paired comparison, 283 and 188 of which were known and novel miRNAs, respectively (Table S5).

Similarly, we used DESeq2 to identify differentially expressed (DE) mRNAs among the six paired sample groups, with threshold criteria of | log2 (Fold Change) | > 1 and FDR < 0.05. We accordingly detected 8940, 10,689, 12,525, 8007, 9807, and 3004 DEmRNAs in the Ov1-vs-Ov2, Ov1-vs-Ov3, Ov1-vs-Ov4, Ov2-vs-Ov3, Ov2-vs-Ov4, and Ov3-vs-Ov4 comparisons, respectively, indicating a greater abundance than the corresponding DEmiRNAs (Fig. 5B). As with DEmiRNAs, we found that the longer the interval between the two sample groups, the greater was the number of detected DEmRNAs.

We used Target Finder to screen hazel mRNA transcripts having high complementary sequence with both the predicted known and novel miRNAs, and accordingly identified 18,137 mRNA targets of the predicted miRNAs. In order to obtain annotation information for the target genes, we used BLAST software to compare the predicted target gene sequences with sequences in the NR, Swiss-Prot, GO, COG, KEGG, KOG, and Pfam databases. We accordingly succeeded in annotating 15,412 of the targeted genes (Table S6). To identify the most biologically relevant miRNA/target pairs, we selected all interacting pairs of DEmiRNAs and target DEmRNAs, resulting in subsets of 1042, 1325, 1653, 894, 947, and 54 DEmiRNA/target DEmRNA pairs for the Ov1-vs-Ov2, Ov1-vs-Ov3, Ov1-vs-Ov4, Ov2-vs-Ov3, Ov2-vs-Ov4, and Ov3-vs-Ov4 comparisons, respectively (Fig. 5C). These DEmiRNA/target DEmRNA pairs and the corresponding annotations are listed in Table S7.

**Fig. 6:**
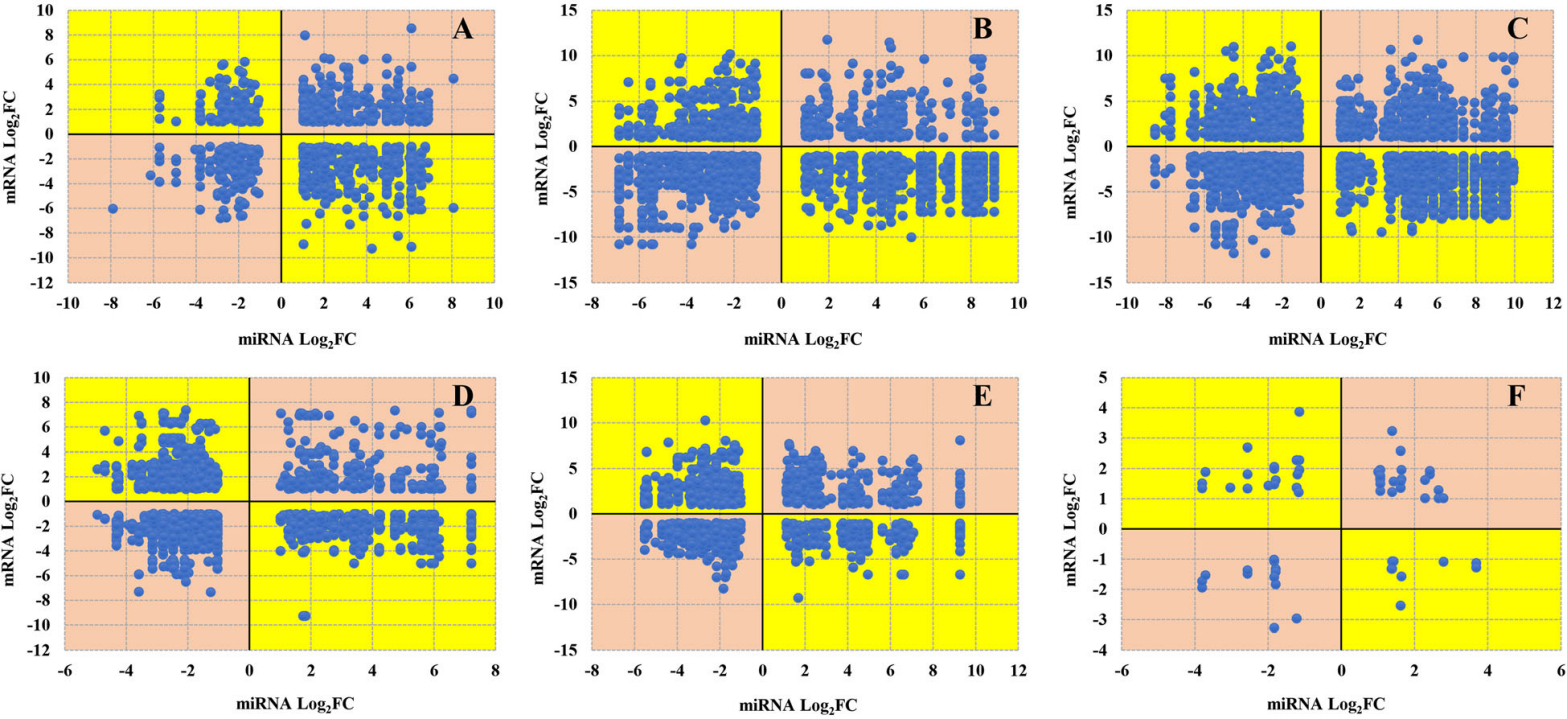
Comparison pairs of differentially expressed miRNAs (DEmiRNAs) and differentially expressed mRNAs (DEmRNAs). DEmRNAs/miRNAs pairs of Ov1-vs-Ov2 (**a**), Ov1-vs-Ov3 (**b**), Ov1-vs-Ov4 (**c**), Ov2-vs-OV3 (**d**), Ov2-vs-Ov4 (**e**) and Ov3-vs-Ov4 (**f**)

Changes in the expression of each DEmiRNA and its target DEmRNAs are shown in Fig. 6. We consequently found relatively similar numbers of negative and positive correlations for DEmiRNA/DEmRNA expression. The two-dimensional plane of our scatter plots was divided into four quadrants by an orthogonal coordinate axis, with data points in the first and third quadrants (with the pink background in Fig. 6) indicating that the DEmiRNAs and target DEmRNAs were both simultaneously up- or downregulated, whereas data points in the second and fourth quadrants (with the yellow background in Fig. 6) indicated that DEmiRNAs and target DEmRNAs showed opposite change trends (i.e., when a DEmiRNA was downregulated its target mRNA was upregulated, and vice versa). In total, 471 DEmiRNAs and their 3117 target DEmRNAs formed 11,199 DEmiRNA/DEmRNA pairs in our six paired comparisons, with each DEmiRNA promoting the differential expression of an average of 6.62 target mRNAs. If an miRNA cleaves its target mRNA, the expression of the target mRNA is expected to decrease, and thus we were particularly interested in the 6230 annotated target DEmRNAs showing an expression trend opposite to that of their targeting DEmiRNA (Table S8).

### Analysis of the target DEmRNA of DEmiRNAs

**Fig. 7:**
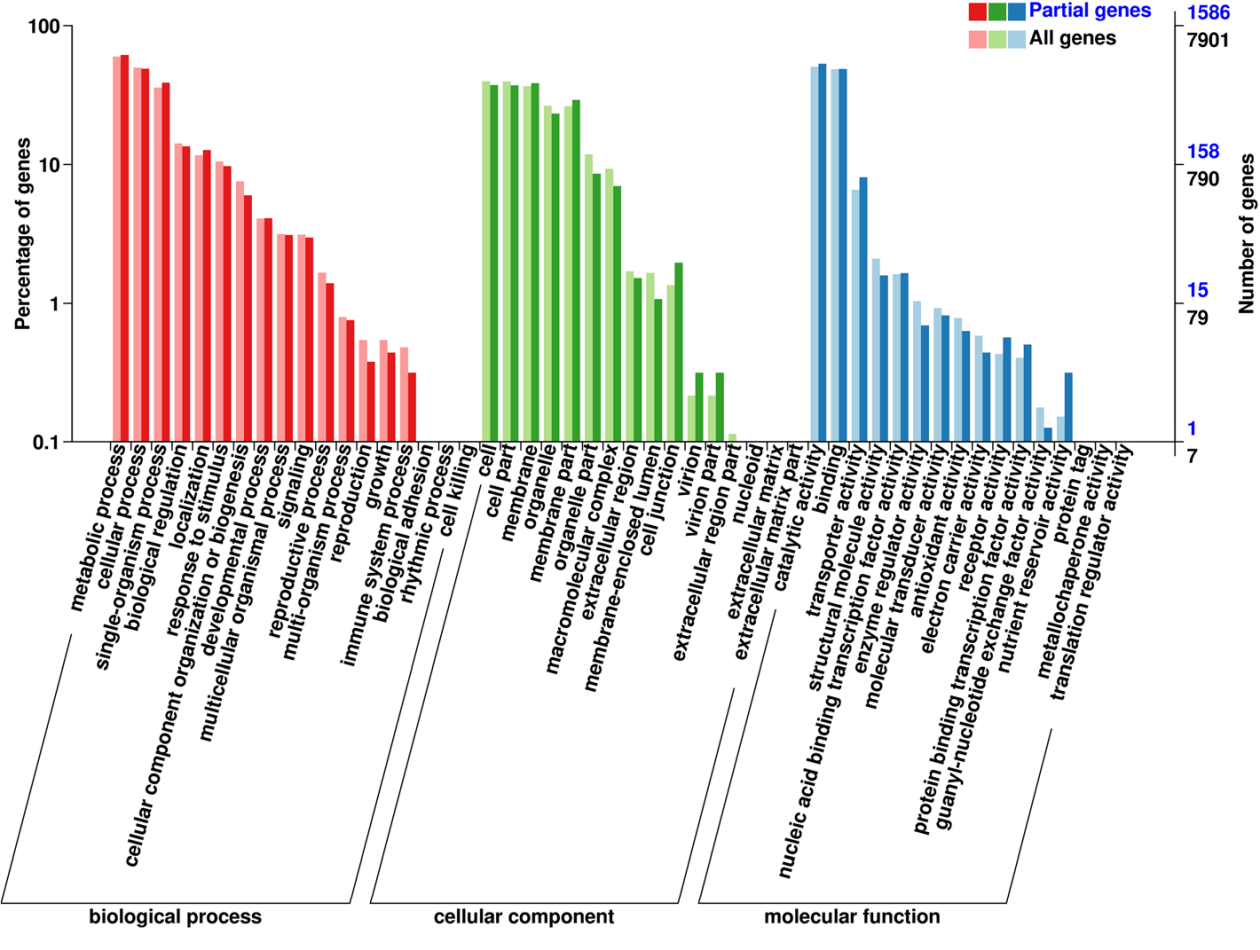
Gene Ontology (GO) classification of all target DEmRNAs of DEmiRNAs. Note: All genes, all target mRNA of miRNA; Partial genes, target DEmRNAs of DEmiRNAs; X-axis shows GO terms; y-axis shows the percentages of target mRNA and target DEmRNAs of DEmiRNAs (number of a particular target mRNA divided by target mRNA or number of a particular target DEmRNAs of DEmiRNAs divided by all target mRNA)

We found that a large number of DEmRNAs targeted by DEmiRNAs encode important transcription factors involved in developmental regulation, including, but not limited to, members of the families bHLH, WRKY, ARF, MYB, and MADS. In order to assess the biological function of the DEmRNA targets of DEmiRNAs, we performed gene ontology (GO) classification analysis using the BMKCloud platform. All 3117 target DEmRNAs with GO annotation identified from the six paired comparisons were assigned to one of three categories, namely, biological process, cellular component, and molecular function (Fig. 7). The majority of the mRNA targets of identified miRNAs were found to be associated with the molecular functions of catalytic activity, binding, transporter activity, structural molecule activity, nucleic acid binding transcription factor activity, enzyme regulator acidity, or molecular transducer activity, and play roles in metabolic processes, cellular processes, biological regulation, localization, responses to stimuli, cellular component organization or biogenesis, development processes, multicellular organismal processes, signaling, or reproductive processes. Most of the protein products of the DEmRNAs are typically located in the cell, cell parts, or organelle membranes. The results of GO enrichment analysis of all target DEmRNAs of all DEmiRNAs (Table S9) indicated that the GO terms of amino acid transmembrane transporter activity (GO:0015171), lipid binding (GO:0008289), and ATP binding (GO:0005524) were mostly significantly enriched in the molecular function category; integral component of membrane (GO:0016021), intracellular membrane-bounded organelle (GO:0043231), and plasmodesma (GO:0009506) were mostly significantly enriched in the cellular component category; and amino acid transmembrane transport (GO:0003333), developmental process (GO:0032502), protein phosphorylation (GO:0006468), carbohydrate transport (GO:0008643), and fatty acid biosynthetic process (GO:0006633) were the most enriched in the molecular function category.

**Fig. 8:**
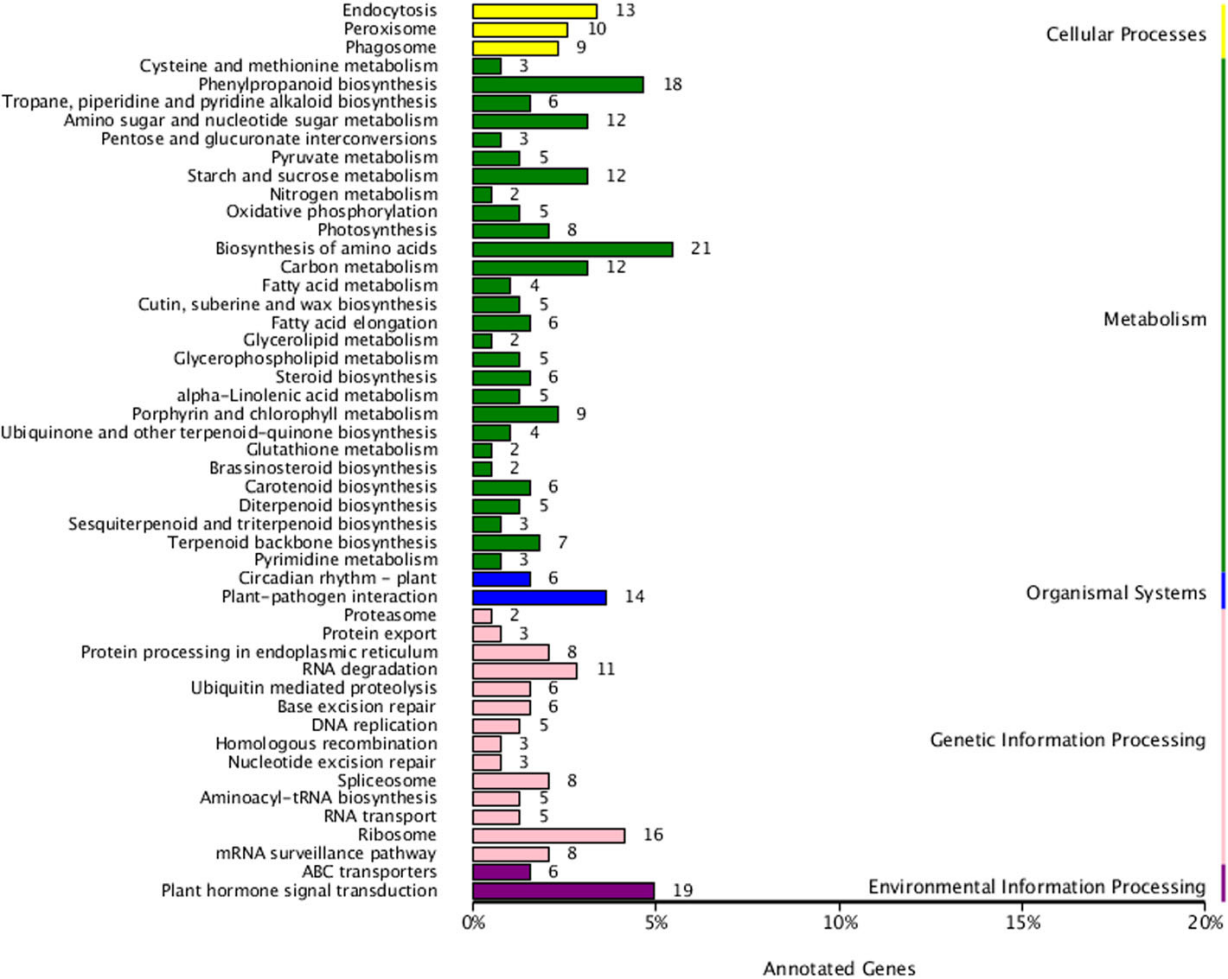
Kyoto Encyclopedia of Genes and Genomes (KEGG) classification of target DEmRNAs of DEmiRNAs. Note: The Y-axis is the name of KEGG pathway, and the X-axis is the number of genes annotated to the pathway and the proportion of the number of genes annotated to the total number of genes annotated

Using classification analysis, we found that the KEGG pathways of all target DEmRNAs were assigned to the following five categories: cellular processes, metabolism, organismal systems, genetic information processing and environmental information processing (Fig. 8). Among these categories, metabolism was the most highly enriched in terms of KEGG pathways and target DEmRNAs. Within this category, biosynthesis of amino acids (ko01230) was the most highly enriched, followed by phenylpropanoid biosynthesis (ko00940), amino sugar and nucleotide sugar metabolism (ko00520), starch and sucrose metabolism (ko00500), carbon metabolism (ko01200), fatty acid metabolism (ko01212), and fatty acid elongation (ko00062). With regards to the genetic information processing category, members in RNA degradation pathway (ko03018) were the most abundant, whereas in the environmental information processing category, the largest number of DEmRNAs were assigned to plant hormone signal transduction (ko04075). When we subsequently performed KEGG enrichment analysis of the DEmRNA targets of DEmiRNAs relative to all target mRNAs of all miRNAs (Table S10), we identified 29 significantly enriched KEGG pathways among the six paired comparisons, including protein export (ko03060), fatty acid elongation (ko00062), starch and sucrose metabolism (ko00500), fatty acid biosynthesis (ko00061), and amino sugar and nucleotide sugar metabolism (ko00520). These results accordingly indicate that vigorous protein, polysaccharide, starch, and fat biosynthesis and transport are necessary for ovule development and growth.

### Validation of DEmiRNAs and their target DEmRNAs by qRT-PCR analysis

**Fig. 9:**
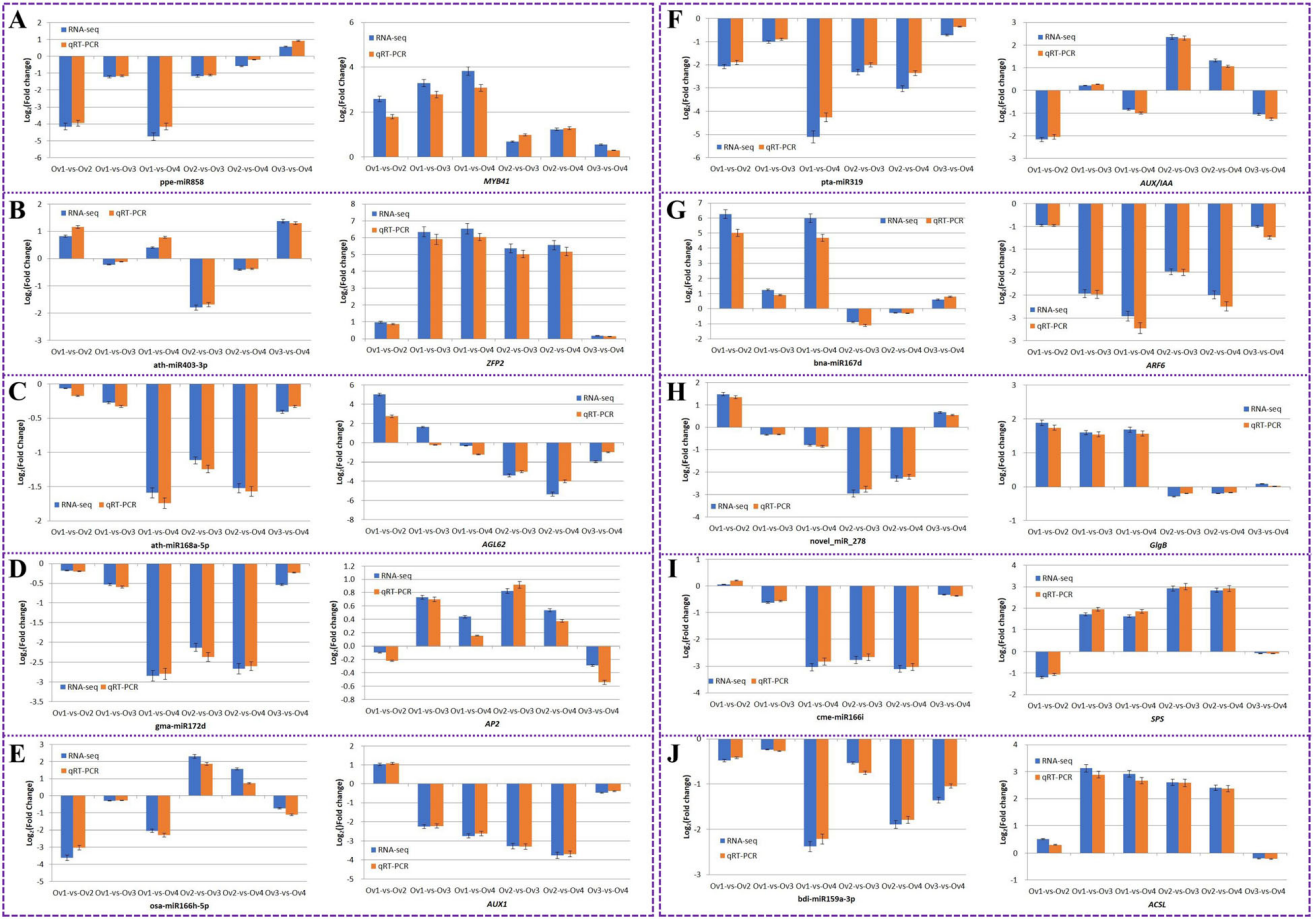
Validation of DEmiRNAs and their target DEmRNAs by qRT-PCR analysis. Bars represent mean ± standard deviation (*n* = 3). The significance level was set as *P* < 0.05

From among the DEmiRNAs and their target DEmRNAs identified in the six paired comparisons, we selected the following 10 DEmiRNA/DEmRNA interacting pairs of interest that may be involved in the regulation of ovary development and growth for validation by qRT-PCR analysis: ppe-miR858/*MYB41*, ath-miR403-3p/*ZFP2*, ath-miR168a-5p/*AGL62*, gma-miR172d/*AP2*, osa-miR166h-5p/*AUX1*, pta-miR319/*AUX/IAA*, bna-miR167d/*ARF6*, novel_miR_278/*GlgB*, cme-miR166i/*SPS* and bdi-miR159a-3p/*ACSL*. In general, we found that the Log_2_(fold change) values obtained based on Illumina sequencing were consistent with those acquired from qRT-PCR (Fig. 9), thereby indicating that the sequencing results we obtained for miRNAs and mRNAs were reliable.

## Discussion

By mining genomic data in silico, 57 putative miRNAs were predicted and identified in *C. avellana* ‘Jefferson’ (OSU 703.007) [22–24]. In the European hazel genome, miR171, miR396, miR482, and miR2118 families were found as in silico expressed putative miRNAs [23]. These findings elucidate the role of miRNAs and their targets in hazel development. In this study, we identified 970 miRNAs at four ovule developmental stages in hazel using the Illumina sequencing platform, of which 766 were known, and 204 were novel miRNAs. All of these miRNAs could be assigned to 165 families. We identified 40, 37 and 38 members in the miR171, miR396 and miR482 families, respectively, suggesting that our results are consistent with the in silico miRNA prediction of the European hazel genome [23]. Clearly, the in silico quantity of miRNA was greater than the available miRNAs in the European hazel genome, which was beneficial for understanding the regulatory role of miRNAs in hazel growth and development. We examined miRNA expression profiles at four developmental stages of hazelnut ovules, and this is the most comprehensive analysis of miRNA profiles during ovule development in hazelnut.

### DEmiRNAs involved in stress tolerance

In plants, endogenous miRNAs play important roles as regulators of gene expression, and are undoubtedly implicated in the regulation of important biological processes, including growth, reproduction, development, differentiation, signal transduction, and defense responses [25, 26]. In this study, we initially focused on a set of DEmiRNAs that were highly expressed in ovules, which may imply their critical regulatory roles in the development of these organs. We accordingly found that these most highly expressed DEmiRNAs are mainly associated with stress tolerance, embryo and seed development, and cell proliferation. The miRNA families containing the most highly expressed DEmiRNAs were MIR858, MIR403 and MIR482, and this set of DEmiRNAs were identified as being involved in stress tolerance [27, 28]. In general, the expression of this set of DEmiRNAs was found to be relatively higher at stages Ov1 and Ov2 than at stages Ov3 and Ov4. In *Arabidopsis*, overexpression of miR858 promotes susceptibility to pathogen infection, whereas the inhibition of miR858 activity via target mimics (MIM858 plants) can confer disease resistance [29]. In paired comparison of Ov1-vs-Ov4, we observed that ppe-miR858 had undergone a 4.79-fold downregulation, whereas its three target genes g15267, g27720, and g33596, which encode the transcription factors *MYB41*, *MYB3*, and *MYB108*, respectively, were upregulated by 5.01-, 1.41-, and 1.08-fold, respectively. In *Arabidopsis*, *AtMYB41* is known to regulate transcriptional and metabolic responses to osmotic stress [30], whereas *AtMYB101* plays a role in controlling pollen tube–synergid interactions during fertilization [31]. In poplar, the transcription factor *PtrMYB3* participates in the regulation of secondary wall biosynthesis [32], and in cotton, *MYB108* is involved in the defense response against *Verticillium dahliae* infection [33]. The diameter of the hazelnut ovule increase rapidly at the Ov3 and Ov4 stages of development (Fig. 1), and accordingly there is a large increase in demand for transport of mineral elements and carbohydrates to the ovule. In hazel, we deduced that the expression of *MIR858* suppressed development due to nutrient competition, resulting in the highly upregulated expression of stress tolerance target genes at the Ov3 and Ov4 stage, including the expression of transcription factors *MYB41*, *MYB3*, *MYB108*, which may be beneficial in promoting tolerance adaption in developing ovules. Similarly, MIR403, and MIR482 may also are involved in ovule development regulation though their target mRNA, including zinc finger CCCH domain-containing protein 2 gene (*ZFP2*) [34] and disease resistance protein RPP13 gene (Table S8) [35].

### DEmiRNAs involved in the regulation of embryo/seed development and cell proliferation

According to the floral quartet model (FQM), the identity of the different floral organs is specified during development by quaternary protein complexes comprising MADS-domain proteins [36]. In *Arabidopsis*, agamous-like MADS-box protein AGL62 is expressed exclusively in the endosperm, and encodes a Type I MADS domain protein that functions as a transcription factor. In *agl62* mutants, there is a premature formation of cell walls in the endosperm, indicating that *AGL62* is required for suppression of cellularization during the syncytial phase, and thereby plays an important regulatory role in seed development [37]. Somewhat inconsistently with these observations, the results obtained in the present study revealed that that two *AGL62*-encoding genes (g15301 and g34233) were highly upregulated during ovule development, whereas the miRNAs targeting these genes (i.e., ath-miR159b-5p and ath-miR168a-5p) showed a decreasing expression pattern. *APETALA 2* (*AP2*) is known to regulate the expression of several floral-specific homeotic genes, including *AGAMOUS* [38]. In the present study, we found that during ovule development, the expressional abundance of two *AP2*-encoding genes (g4294 and g15260) upregulated concomitant with the downregulation of targeting DEmiRNAs (i.e., gma-miR172d and csi-miR172c-3p). Hence, high and increasing expression pattern of *AGL62* and *AP2* in ovules is suggestive of their important roles in ovule development. Furthermore, we found that their expression is regulated by MIR159, MIR168, and MIR172; however, further molecular analyses will be necessary to determine whether AP2 regulate the expression of *AGL62*.

Other important developmental regulators are known to be regulated by MiR159, MIR166, MIR396, and MIR319. MiR159 is a highly conserved miRNA that plays roles in short-day flowering, anther development, and seed germination via repression of *GAMYB-like* genes [39]. *MIR166* genes are dynamically controlled in regulation of the shoot apical meristem (SAM) and floral development in parallel with the WUSCHEL (WUS)-CLAVATA (CLV) pathway [40]. It is conceivable that *MIR166* contributes to the induction of somatic embryogenesis via targeting *PHABULOSA*, a positive regulator of *LEAFY COTYLEDON2* that controls embryogenic induction via activation of the auxin biosynthesis pathway [41]. The MIR396 family is known to target members of the Growth-Regulating Factor (GRF) gene family, and ath-miR396 overexpressors or *grf* mutants have been found to be characterized by embryogenic defects such as cotyledon fusion [42]. miR319 regulates transcription factors in the TCP family, and high levels of miR319 or low TCP activity can promote cell proliferation [43]. These DEmiRNAs are predicted to cleave the following target DEmRNAs: *myb domain protein 101* (*MYB101*, g5913), *ABSCISIC ACID INSENSITIVE 5* (*ABI5*, g6983), *GROWTH REGULATING FACTOR5* (*GRF5*, g17778), *GRF12* (g10975), *GRF1* (g13479), *polyadenylate-binding protein C1* (*PABPC1*, g7335), and *T-complex protein 1* (*TCP-1*, g23844). The DEmiRNAs also regulate important biological processes, including seed development, embryogenesis, ovule growth, and cell proliferation. The aforementioned five miRNA families include a number of the DEmiRNAs identified in the present study, each of which is predicted to cleave several differentially expressed target genes, thus forming hundreds of DEmRNAs/DEmiRNA pairs. Collectively, these DEmRNAs/DEmiRNA pairs constitute a complex regulatory network that function to coordinate development of the hazelnut ovule.

### DEmiRNAs associated with the auxin transduction pathway

Our GO and KEGG pathway classification of all target DEmRNAs revealed that multiple DEmRNAs are involved in plant hormone signal transduction (Figs. 7 and 8). Although we identified DEmRNAs in all plant hormone signal transduction pathways, we focused primarily on a group of DEmRNAs/DEmiRNA pairs associated with the auxin (IAA) signal transduction pathway. We found that MIR166 (osa-miR166h-5p) was highly expressed at stage Ov1 of ovule development, whereas its expression was maintained at lower levels from stage Ov2 to Ov4. Its target gene *AUX1*, encoded by g30973, was identified to be significantly unregulated in the Ov1-vs-Ov2 pairwise comparison, indicating that auxin influx into the ovule is beneficial for embryo development at stage Ov2.

Aux/IAA proteins are important repressors in auxin signaling pathway. Auxin response factors (AFRs) are responsible for the expression regulation of early auxin response genes, and Aux/IAA can form heterodimer with ARFs and inhibit their transcriptional regulation activity subsequently [44]. We found that the expression of MIR164 (osa-miR164d) and MIR 319 (pta-miR319) was relatively higher at stages Ov1 and Ov2, and lower at stages Ov3 and Ov4. These DEmiRNA target auxin responsive proteins are encoded by g24866 and g29299, respectively, and changes in their patterns of expression were found to be opposite to those of osa-miR164d and pta-miR319.

In *Arabidopsis*, ARF2/MNT (MEGAINTEGUMENTA) is a repressor of cell division and organ growth [45], and arf2 mutants have phenotypes of pleiotropic development, including delayed flowering, senescence and abscission, floral organ malformation and sterility [46, 47]. In *Camellia oleifera*, silencing of *ARF2* using *amiRARF234* was found to promote identity defects in cells at the micropylar pole of embryos and subsequent seed abortion [26]. Furthermore, *ARF6* is known to regulate gynoecium maturation, and the flowers of *arf6 arf8* double mutants are characterized by immature gynoecia in *Arabidopsis* [48, 49]. In this study, we identified four DEGs (g28230, g22071, g14283, and g24866) that encode *ARF6*, *ARF2*, *ARF17*, and *ARF9*, respectively. Among these, ARF6 and ARF2 are predicted to be cleaved by dozens of DEmiRNAs belonging to the MIR167_1, miR159, and miR319 families. The expression abundance of *ARF6* and *ARF2* deceased and increased along with development, respectively. *ARF6* is targeted by DEmiRNAs of the MIR167_1 family (e.g., bna-miR167d, vvi-miR167c, and ccl-miR167a), the expression of which tended to increase with ovule development. Conversely, the expression of miR159 and miR319 family DEmiRNAs targeting *ARF2* (e.g., sof-miR159c, bdi-miR159a-3p, and ath-miR319a) was observed to decrease with ovule development. Taken together, our results indicate that the relatively high expression of DEmiR159 and DEmiR319 and low expression of their target *ARF2* at stage Ov3 and Ov4 contribute to ovule growth and filling, whereas a decrease and increase in the expression of MIR167_1 and its target *ARF6*, respectively, at all four developmental stages indicated their regulatory role in embryo sac maturation and subsequent ovule growth and filling.

Results obtained from histochemical analysis of auxin indicated that auxin is enriched in the growth center of pistillate inflorescences and young ovaries [4]. In this study, we found *AUX1* expression was relatively higher at stage Ov2 than stage Ov3 and Ov4. As active repressors, the expression of Aux/IAA proteins were relatively lower at stages Ov1 and Ov2, whereas expression of *ARF2*, a repressor of cell division and organ growth, increased concomitant with development, and that of ARF6 decreased with development. These results accordingly indicate that auxin signal transduction tends to be active at stages Ov1 and Ov2, which may facilitate the development and differentiation of the embryo sac and embryo at these stages, whereas subsequent inactivation of auxin signal transduction may contribute to promoting the growth and filling of ovules at stages Ov3 and Ov4. Moreover, we found that the expression of this set of important regulators was strictly regulated by multiple miRNA families, including MIR164, MIR 319, MIR167_1, miR159, and miR319.

### DEmiRNAs participate in the regulation of carbohydrate, protein, and lipid biosynthesis

GlgB, a 1, 4-alpha-glucan branching enzyme, is a key enzyme in the starch synthesis pathway, in which it catalyzes the formation of the alpha-1, 6-glucosidic linkages in glycogen by cleaving 1, 4-alpha-linked oligosaccharides from growing alpha-1, 4-glucan chains and subsequently attaching these oligosaccharide to the alpha-1, 6 position [50]. In our Ov1-vs-Ov2 and Ov1-vs-Ov3 comparative analyses, we observed that *GlgB* (g8185) was upregulated by 1.82- and 1.79-fold, respectively, indicating a pattern of increasing expression with development. *GlgB* is the target gene of the novel miR_278, which was found to be downregulated by 1.59-fold in the Ov1-vs-Ov3 pairwise comparison. Sucrose-phosphate synthase (SPS) plays a major role in photosynthetic sucrose synthesis by catalyzing the rate-limiting step of sucrose biosynthesis from UDP-glucose and fructose-6-phosphate, and has been demonstrated to play a role in regulating carbon partitioning in plant leaves [51]. In our Ov1-vs-Ov3, Ov1-vs-Ov4, Ov2-vs-Ov3, and Ov2-vs-Ov4 comparisons, we found that SPS, which is encoded by g751, was upregulated by 2.55-, 2.69-, 3.39-, and 3.54-fold respectively, indicating that its expression increased with development. In our analyses, we found that the MIR166 family member cme-miR166i, which is predicted to cleave SPS mRNA, was initially highly expressed in ovules, whereas its expression decreased with ovule development. KEGG pathway enrichment analysis revealed that DEmRNAs were significantly enriched in the starch and sucrose metabolism pathway (ko00500) category (Table S10) for the Ov1-vs-Ov4 comparison. Consistent with these findings, we suggest that increases in the expression of *GlgB* and *SPS* may promote the biosynthesis of starch and carbon partitioning in hazelnut, and that novel miR_278 and cme-miR166i may be involved in these biological process via their regulation of *GlgB* and *SPS*.

KEGG pathway enrichment analysis indicated that DEmRNAs were significantly enriched in several amino acid- and protein-related pathways (Table S10), including protein export (ko03060), arginine and proline metabolism (ko00330), alanine, aspartate and glutamate metabolism (ko00250), and tryptophan metabolism tryptophan metabolism (ko00380). Glutamate synthase (NADH) (GLT1), encoded by g3579, is a key enzyme in ammonia assimilation processes, generating l-glutamate from l-glutamine and 2-oxoglutarate, and represents an alternative pathway to l-glutamate dehydrogenase for the biosynthesis of l-glutamate [52]. In the Ov2-vs-Ov3 comparison, GLT1 was found to be upregulated by 1.38-fold, which may contribute to promoting nitrogen assimilation at stage Ov3. Although hbr-miR9386 is predicted to cleave g3579, we found that it was significantly upregulated in the Ov2-vs-Ov3 comparison. Moreover, its sequence started with base T instead of U, which is not a typical mature miRNA feature. These results thus indicate that upregulation of GLT1 may contribute to the acceleration of protein biosynthesis in hazelnut; however, its expression may not necessarily be regulated by miRNAs.

The conversion of acetyl CoA to malonyl CoA, catalyzed by acetyl-CoA carboxylase (ACCase), is an initial step in fat synthesis [53]. The conversion from hexadecanoic acid to hexadecanoyl-CoA is catalyzed by long-chain acyl-CoA synthetase (ACSL) [54], and hexadecanoyl-CoA subsequently undergoes further modification in fatty acid elongation. In the Ov2-vs-Ov4 comparison, we found that DEmRNAs were significantly enriched in the Fatty acid biosynthesis (ko00061) KEGG pathway, and that ACCase, encoded by *g121*, was upregulated by 2.41-fold, whereas its targeting miRNA (novel miR_278) was downregulated by 2.17-fold. Furthermore, we found that *g20632* and *g8996*, which encode ACSL, were upregulated by 1.09- and 2.90-fold, respectively, whereas the miR159 family miRNA bdi-miR159a-3p, which targets both these genes, showed a decrease in expression with ovule development, being downregulated by 1.81-fold in the Ov2-vs-Ov4 comparison. Taken together, ACCase and ACSL are key enzymes in the initial stages of fat biosynthesis and the final step prior to fatty acid elongation, respectively, and all the three DEmRNAs were upregulated in the Ov2-vs-Ov4 comparison, concomitant with a downregulation of their targeted DEmRNAs. These DEmRNA/DEmiRNA pairs may be responsible for the activated lipid biosynthesis at stage Ov3 and Ov4.

## Conclusions

We identified a total of 970 miRNAs in hazel, 471 of which were found to be differentially expressed in six paired stage-wise comparisons. Most of the highly expressed DEmiRNAs were found to contribute to stress tolerance, embryo and seed development, and cell proliferation. Among these, we assume that MIR858, MIR403, and MIR482 function to depress development due to nutrient competition, resulting in the high upregulated expression of stress tolerance target genes, including *MYB41*, *MYB3*, *MYB108*, and *ZFP2*. We also deduced that DEmiRNAs within the MIR159, MIR166, MIR167_1, MIR396, and MIR319 family play important roles in seed development, embryogenesis, ovule growth, and cell proliferation via their regulation of *MYB101*, *ABSCISIC ACID-INSENSITIVE 5*, *GRF5*, *GRF12*, *GRF1*, *polyadenylate-binding protein gene*, *TCP-1*, and *AP2.* Similarly, we suggest that auxin signal transduction may be regulated by DEmiRNAs such as miR159 and miR319 in the MIR164, MIR 319, MIR167_1 families, whereas novel miR_278 and cme-miR166i may be involved in starch and sucrose metabolism pathways via their regulatory roles related to *GlgB* and *SPS* expression. Moreover, novel miR_278 and bdi-miR159a-3p may participate in fat biosynthesis via their regulatory roles related to ACCase and ACSL expression. We believe that the findings of this study provide valuable insights that will contribute to gaining a better understanding of regulatory roles of miRNAs in ovule development in hazelnut.

## Methods

### Plant materials

In 2018, the plant samples used in the present study were collected from a hazel orchard in Siping, Jilin Province, China, in which the major hazelnut cultivar was *Corylus heterophylla × C. avellana* ‘Dawei’, and *C. heterophylla* × *C. avellana* ‘Bokehong’ was used as a pollination cultivar. Pollen of ‘Bokehong’ was collected and air-dried using the male flower branch culture method, as described previously [6]. These hazel cultivars were subjected to molecular analysis at the College of Life Sciences, Jilin Normal University, using a simple sequence repeat (SSR)-based technique and seven primer pairs, as described in our previous study [4, 5]. In the orchard, the two cultivars are arranged at regular intervals, with row and plant spacing of 2.0 m and 3.0 m, respectively. The hazel trees are 12 years old and 3.0 to 3.5 m in height, and the local hazel blooming and nut harvest dates are approximately April 20 and August 25, respectively. In total, 60 trees of ‘Dawei’ were chosen and used as our study material, consisting of three biological replicates of 20 trees each. On April 10, about 3000 quality pistillate inflorescences of ‘Dawei’ were randomly bagged and tagged, and each chosen tree had about 40 tagged inflorescences. On April 18 (blooming date), artificial pollination was carried out to exclude the possibility of self-pollination. Sampling were collected on May 20, June 20, July 20, and August 20, respectively, which corresponded to the stages of ovule formation (Ov1 stage, Fig. 1A), early ovule growth (Ov2 stage, Fig. 1B), rapid ovule growth (Ov3 stage, Fig. 1C), and ovule maturity (Ov4 stage, Fig. 1D), respectively. Ovules within the ovaries were isolated manually and stored in liquid nitrogen for further RNA extraction. For all isolated ovules from the same developmental stage, only medium ovules were used as study material for sample homogeneity. Scientific research activities including sampling were approved by the owner of hazel garden. The voucher specimens of these materials were publicly deposited in Shenyang Agriculture University, Shenyang, China. All field experiments were performed in accordance with the Convention on the Trade in Endangered Species of Wild Fauna and Flora. Using the collected ovules mentioned above, we constructed 12 digital gene expression (DGE) profiling libraries in order to investigate changes in gene expression during the four developmental stages, including Ov1A, Ov1B, Ov1C, Ov2A, Ov2B, Ov2C, Ov3A, Ov3B, Ov3C, Ov4A, Ov4B, and Ov4C. For the library names, ‘Ov1’, ‘Ov1’, ‘Ov1’, and ‘Ov1’ indicate four sequential ovule developmental stages and ‘A’, ‘B’, and ‘C’ indicate the three biological replicates.

### Small RNA sequencing and data processing

Total RNA was extracted using a Plant Total RNA Kit (Sigma-Aldrich, St. Louis, MO, USA) according to the product instructions. Purity and concentration of RNA were determined by a spectrophotometer (NanoDrop, 2000), and integrity of total RNA was assessed on a 1% denaturing formaldehyde-agarose gel. Good quality RNA was used to construct RNA libraries using an NEB Next Ultra small RNA Sample Library Prep Kit for Illumina (New England BioLabs, Ipswich, MA, USA). Initially, 3′ and 5′ adaptor sequences were ligated to the total RNA, and cDNA was obtained after reverse transcription. Thereafter, PCR amplification and gel purification were performed to generate the small RNA libraries, and single-end 50 nt (SE50) sequencing were performed using a HiSeq 4000 sequencing platform (Illumina, San Diego, CA, USA). Raw small RNA sequencing and mRNA data of 12 samples at four developmental stages were deposited in the Sequence Read Archive (https://www.ncbi.nlm.nih.gov/sra/?term=PRJNA591492). The raw sequencing reads were filtered to generate clean reads using Trimmomatic software [55], by excluding the following reads: (1) reads with more than 20% of bases less than Q30, (2) reads with more than 10% unknown bases, (3) reads without an 3′ adaptor, and (4) reads longer than 30 nt and shorter than 18 nt. The clean reads were compared against the Silva, GtRNAdb, Rfam, and Repbase databases using Bowtie software (version v1.0.0) [56], and parameters were set to default setting. Using Bowtie software [56], non-coding RNAs, such as ribosomal RNAs (rRNAs), transporting RNAs (tRNAs), intranuclear small RNAs (snRNAs), nucleolar small RNAs (snoRNAs), and repetitive sequences, were removed to obtain unannotated reads containing microRNA, and these unannotated reads were aligned with the reference Jefferson hazelnut genome (https://www.cavellanagenomeportal.com).

### miRNA identification

Sequences mapped to reference genome were aligned with the known precursor sequences in the miRBase database using the miRDeep2 (Plant) software package (version v2.0.5) [19] to determine their expression, and parameters were set to default setting. The Pearson correlation coefficient was used to quantify similarities between samples using the following formula [57]:

$$ {\rho}_{X,Y}=\frac{co\upsilon \left(X,Y\right)}{{}^{\sigma }{X}^{\sigma }Y}=\frac{E\left(\left(X-{\mu}_X\right)\left(Y-{\mu}_Y\right)\right)}{{}^{\sigma }{X}^{\sigma }Y}=\frac{E(XY)-E(X)E(Y)}{\sqrt{E\left({X}^2\right)-{E}^2(X)\sqrt{E\left({Y}^2\right)-{E}^2(Y)}}} $$

Putative precursor sequences were obtained by comparing the positional information in the hazel genome with the mapped reads. On the basis of read distribution information regarding the precursor sequences (mature, star, loop) and the precursor structural energy information (RNA fold randfold), we used a Bayesian model to identify novel microRNAs [58].

### miRNA differential expression analysis

Expression data were obtained for the miRNAs in each sample, and the miRNA expression levels were normalized as tags per million (TPM) using following formula [59]: TPM = read count × 1,000,000/mapped reads, where read count and mapped reads are the number of reads aligned to a single miRNA and reads aligned to all miRNAs, respectively. DESeq2 (version 1.6.3) [60] was used to analyze the differential expression of miRNAs among the sample groups and to determine the differentially expressed miRNAs (DEmiRNA), where | log_2_(FC) | > 1 and a false discovery rate (FDR) < 0.05 were used as discrimination criteria. Fold change (FC) represents the ratio of expression between two sample groups. Comparisons were performed between each pair of sample libraries (Ov1-vs-Ov2, Ov1-vs-Ov3, Ov1-vs-Ov4, Ov2-vs-Ov3, Ov2-vs-Ov4, and Ov3-vs-Ov4), in which the former was used as the control and the latter as the experimental group in each paired comparison. The six pairs of miRNA profiles were compared in order to determine the changes in miRNA expression during ovule development and growth in hazel.

### Prediction of miRNA target genes and their annotation

On the basis of known and newly predicted miRNAs and gene sequence information for corresponding species, we used Target Finder software (version v1.6) [61] to predict target genes using default parameters. BLAST software (version v2.2.26) was then used to align the predicted target gene sequences of DEmiRNAs to NR [62], Swiss-Prot [63], GO [64], COG [65], KEGG [66], KOG [67], and Pfam [68] databases to obtain annotation information.

### Correlation of miRNA/target mRNA expression

A paired design of mRNA extraction, mRNA library construction, and sequencing of the aforementioned 12 samples was carried out as described previously [4, 5], sequencing both the miRNA and mRNA fractions obtained for each biological replicate. On the basis of the mRNA expression results, differentially expressed genes (DEGs) were identified using DESeq2 (version 1.6.3) [60] by setting the threshold of fold change as > 2.00 and FDR as < 0.05. The Pearson correlation coefficient of the miRNA/target pair for each DEG was calculated using normalized expression data in terms of RPM (miRNA reads per million mapped reads) and fragments per kilobase per transcript per million mapped reads (FPKM).

### Analysis of miRNA gene targets

Using the online Gene Ontology (GO) and KEGG tools of BMKCloud (http://www.biocloud.net), GO and KEGG classification and enrichment analysis of all identified miRNA target gene and DEmiRNA-targeted mRNA with GO terms was conducted. The threshold for significant enrichment was set as *P* < 0.05.

### Real-time quantitative PCR analysis of miRNAs and predicted target gene

The total RNA samples used in sequencing were also subjected to real-time quantitative RT-PCR of miRNAs and predicted target genes. The primer pairs of several target genes used for real-time quantitative RT-PCR were designed using Primer 5.0 (http://downloads.fyxm.net/Primer-Premier-101178.html) and synthesized by Sangon Biotech Co, Ltd. (Shanghai, P.R. of China). The real-time quantitative RT-PCR analysis of mRNA was performed as described previously [4, 5]. We selected 10 DEmiRNAs of interest and their respective target DEmRNAs for real-time quantitative PCR (qPCR) analysis of each comparison. Reverse transcription of mature miRNA was performed using an miRNA First-Strand cDNA Synthesis Kit, and miRNA qRT-PCR were carried out using an miRNA Real-Time PCR assay kit and a Stratagene Mx 3000P qPCR system (Agilent Technologies, Santa Clara, CA, USA). The two kits mentioned above were purchased from Aidlab Biotechnologies, Beijing, China. Following the instructions of these kits, we carried out experiments of reverse transcription and miRNA qRT-PCR, and set all parameters of qRT-PCR. All reactions of qRT-PCR included three biological repeats, and each biological repeat included three technique repeats. U6 was used as internal control for miRNA, and the 2^−ΔΔCT^ method was applied to calculate the relative changes in gene expression [69].

## Supplementary information

**Additional file 1 Figure S1** Sample correlation diagram

**Additional file 2 Table S1** All microRNAs and their expression

**Additional file 3 Table S2** Analysis of miRNA family of all identified miRNA

**Additional file 4 Table S3** Target mRNA of all identified miRNA

**Additional file 5 Table S4** All miRNA count at four developmental stages

**Additional file 6 Table S5** All DEmiRNA expression at four developmental stages

**Additional file 7 Table S6** Annotation of all target mRNA

**Additional file 8 Table S7** DEmRNAs of DEmiRNAs

**Additional file 9 Table S8** Target DEmRNA of DEmiRNA showing opposite changing trend

**Additional file 10 Table S9** GO enrichment of DEmRNA of DEmiRNA

**Additional file 11 Table S10** KEGG enrichment of DEmRNA of DEmiRNA

**Additional file 12 Table S11** Primers for qRT-PCR analysis

## Data Availability

Data are available in additional files, and materials are available from the authors upon request. Raw small RNA sequencing and mRNA data of 12 samples at four developmental stages were deposited in the Sequence Read Archive (https://www.ncbi.nlm.nih.gov/sra/?term=PRJNA591492). microRNA data have 12 consecutively numbered SRA numbers using the NCBI link, and they are from SRX7212427 to SRX7212438. mRNA data have 12 consecutively numbered SRA numbers using the NCBI link, and they are from SRX7224620 to SRX7224631, and the data under the every SRA number contain both mRNA and long-chain non-coding RNA data.

## Abbreviations

ACCase: Acetyl-CoA Carboxylase
ACSL: Long-chain acyl-CoA synthetase
AGL: Agamous-like MADS-box protein
GRF: Growth-regulating factor
AP: APETALA
ARF: Auxin response factor
AUX/IAA: Auxin/INDOLE-3-ACETIC ACID
AUX: Auxin influx transporter AUX
DEmiRNA: Differentially expressed miRNA
DEmRNA: Differentially expressed mRNA
GlgB: 1,4-alpha-glucan branching enzyme
GLT1: Glutamate synthase1 (NADH)
miRNA: MicroRNA
MYB: Transcription factor MYB
SPS: Sucrose-phosphate synthase
TCP: Transcription factors TCP
ZFP2: CCCH domain-containing protein 2
