# Supplementary figures and images for: Identification of vital candidate microRNA/mRNA pairs regulating ovule development using high-throughput sequencing in hazel
Source: BMC Dev Biol. 2020 Jul 1;20:13. doi: 10.1186/s12861-020-00219-z (PMC7329476; doi:10.1186/s12861-020-00219-z)

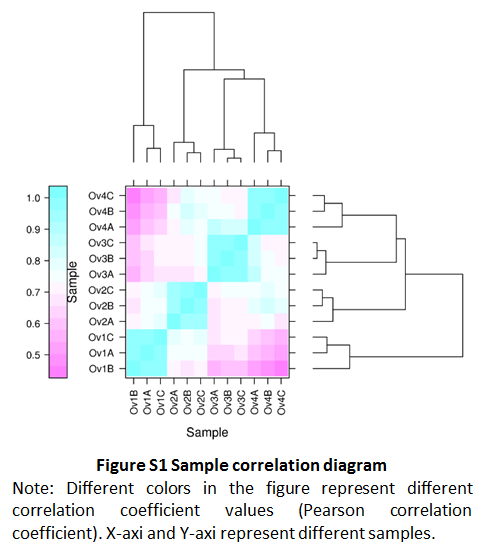

Supplement: Supplementary file 1 — Additional file 1 Figure S1 Sample correlation diagram [file 12861_2020_219_MOESM1_ESM.tif]
